# Urinary schistosomiasis and the associated bladder cancer: update

**DOI:** 10.1186/s43046-020-00055-z

**Published:** 2020-11-30

**Authors:** Mohamed S. Zaghloul, Tarek M. Zaghloul, Mai K. Bishr, Brian C. Baumann

**Affiliations:** 1 Radiation Oncology Department, National Cancer Institute, Cairo University, Fom El Khalig Square, Cairo, Egypt; 2 Surgical Oncology Department, National Cancer Institute, Cairo University, Fom El Khalig Square, Cairo, Egypt; 3 University College London Hospitals NHS Foundation Trust, London, UK; 4 Department of Radiation Oncology, Washington University in St. Louis, St. Louis, USA

**Keywords:** Schistosomiasis, Bilharziasis, Schistosoma-associated bladder cancer (SA-BC), Carcinogenesis, Treatment

## Abstract

**Background:**

Urinary schistosomiasis and its severe complications, mainly bladder cancer, are scarce in non-endemic areas. The deficiency in knowledge and clinical experience of schistosomiasis may lead to inadequate management. Highlighting these topics may be of value, especially with the increased immigration from endemic low-/middle-income countries (LMIC) to non-endemic high-income countries (HIC). Schistosomiasis is a parasitic infection endemic in many low- and middle-income countries. It can affect various systems but is best known for its effect on the urinary system.

**Main Body:**

PubMed, Scopus, Google Scholar, and the Cochrane Library databases were searched for urinary schistosomiasis and its related bladder cancer published from 1980 till 2020.

*Schistosoma haematobium* (SH) infecting the urinary bladder was considered by the IARC as group 1 definitive biological carcinogenic agent. Several carcinogenic pathways have been postulated but the exact mechanism(s) are not defined yet. A more thorough understanding of the parasite life cycle was explored to help eradicate the infection especially for the immigrants from endemic areas. This may prevent or slow down the process of carcinogenesis that leads to Schistosoma-associated bladder cancer (SA-BC), which is usually, but not conclusively, squamous cell carcinoma. Treatment of SA-BC generally follows the same guidelines as urothelial Schistosoma-non-associated bladder cancer (SNA-BC) management; however, prospective trials to confirm and refine the treatment approach for SA-BC have been relatively limited.

**Conclusion:**

The available data showed that despite some etiologic and carcinogenic differences, the oncologic outcomes are generally comparable for SA-BC and NSA-BC when adjusting for stage, risk status, and comorbidities.

## Background

The world is recently facing a significant migratory flow from Africa to Europe. Many European countries received thousands of immigrants along the Mediterranean basin. This resulted in an increased incidence of tropical diseases, including the tropical urological ones, that were previously considered uncommon in the European countries [1]. Salas-Caronas et al. [2] underwent a study on 3090 sub-Saharan immigrants in Spain and found that 10.5% of them having confirmed schistosomiasis and 3.5% presented with severe urological complications. This made the European Health Service authorities recommended the use of serological screening in the new immigrants from Schistosoma-endemic countries [3]. Furthermore, Mantico et al. [4] underwent a survey study that was participated by 109 African and 193 European urologists. The authors discovered a significant deficiency in knowledge of tropical urological diseases by the European urologists compared to the Africans. Marked superior knowledge of European urologists who previously worked in developing countries was documented. The 22-item survey included specific questions about clinical features, diagnosis, and management of tropical urological diseases. The lack of knowledge may lead to misdiagnosis, redundant special investigations, or incorrect managements. This deficiency may result in patient harm and/or wasting of the healthcare resources.

The main aim of this review is to present an update of the known knowledge of schistosomiasis, the uncommon disease in Europe, to help spread knowledge and remove the ambiguity of the disease to help proper management and avoid cost overruns.

## Main text

After approval of the Egyptian National Cancer Institute IRB, all publications (1980–2020) about urinary schistosomiasis and Schistosoma-associated bladder cancer (SA-BC) were thoroughly reviewed, covering all aspects of schistosomiasis, its carcinogenesis, clinical presentations, complications, and treatment. MEDLINE (PubMed), Scopus, Google Scholar, and the Cochrane Library databases were searched using the keywords “Schistosomiasis, Bilharziasis, carcinogenesis, management, Schistosoma- associated bladder cancer, SA-BC.” Only English literature was assessed.

### Historical background

Schistosomiasis has long been a public health scourge. The disease was first described in the papyri of ancient Egypt, and the ancient Egyptian mummies have been found to contain calcified parasite eggs. The first modern description of urinary schistosomiasis was reported by a German physician working at the Kasr-El-Aini Medical School, Cairo University, “Theodor Bilharz” (1825–1862). Bilharz discovered the trematode worm causing urinary schistosomiasis in autopsy studies. Initially named *Distomum haematobium*, it was renamed in his honor as *Bilharzia haematobium*. The name *Schistosoma* (Greek: “split body”) based on the morphology of the male worm was proposed and officially adopted by The International Commission on Zoological Nomenclature [5]. The relationship between schistosomiasis and bladder cancer was first postulated by Ferguson in 1911 at Cairo University School of Medicine, in an autopsy study of Schistosoma-infected patients [6].

### Epidemiology

Schistosomiasis is a parasitic disease caused by the blood fluke infestation. The parasites live in certain species of freshwater snails. The infectious form of the parasite emerges from the freshwater snail and infect humans upon skin contact. Schistosomiasis can affect many organ systems, causing considerable morbidity, including SA-BC.

The World Health Organization (WHO) estimates that schistosomiasis infects more than 200 million people worldwide, including over 76 low- and middle-income countries, in Africa, Asia, and Latin America [7]. Schistosomiasis is recognized as one of the most devastating endemic diseases in tropical areas particularly sub-Saharan Africa. A total of 779 million people is estimated to be at risk of infection while about 250 million people are currently infected [8].

Human schistosomiasis includes 6 species. *S. haematobium* (SH) is implicated in 130 million cases, *S. mansoni* in 73 million cases, *S. japonicum and* S. *mekongi* in two million cases, *S. malayensis* in many cases in Malaysia, and *S. guineensis* and *S. intercalatum* in rain forest areas in central Africa [9, 10]. The mortality rates due to schistosomiasis and their complications have been estimated at 280,000 deaths per year. Schistosomiasis can cause many health problems to the extent that disability-adjusted life years due to schistosomiasis burden exceeds 70 million, worldwide [11]. Despite public health campaigns to eradicate schistosomiasis, it remains a major public health crisis. The disease can cause abdominal pain, diarrhea, portal hypertension, esophageal varices, hepatic periportal fibrosis, ascites, chronic anemia, chronic disability, cognitive impairment, and many other illnesses and complications. The disease is associated with poverty and contaminated river water. One of the well-known late complications of schistosomiasis is bladder cancer with a 2- to 14-fold increase in the risk of its occurrence [12].

Poor communities without access to proper sanitation are mainly the maximum prevalence of Schistosomiasis, particularly in sub-Saharan Africa. The disease affects mainly rural communities, mostly agricultural and freshwater fishing populations. Inadequate hygiene and contact with infected water are the main contributing factors for high-infestation rates, particularly among school-age children [13] In the past, the documented SH prevalence rate in Egypt was 37–48% that decreased due to the successful anti-bilharzial campaign to less than 3% and continue decreasing [14]. Previously, bladder cancer accounted for ~ 27% of the total incidence of cancers in Egypt that subsequently decreased to 11.7 % in recent years [14]. Many studies in Egypt have reported lower rates of SH infection, lower rates of associated epidermoid carcinoma of the bladder, and higher rates of a urothelial type of bladder cancer [15–17].

### The parasite life cycle

**Fig. 1:**
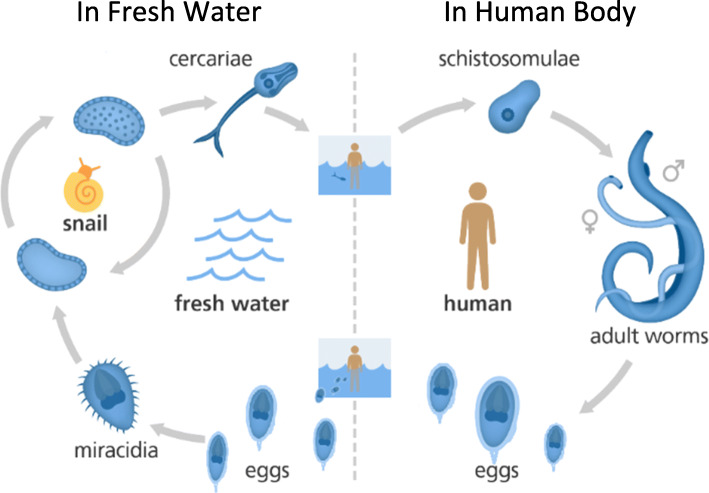
Life cycle of *Schistosoma haematobium* in freshwater (asexual reproduction in the snail) and in the human body (sexual reproduction in genitourinary venules)

Schistosomes are blood flukes, belonging to the genus *Schistosoma*; family, Schistosomatidae; order, Digenea; class, Trematoda; phylum, Platyhelminthes. They have no body cavity and have no specialized circulatory and respiratory organs; however, their flattened shapes allow oxygen and nutrients to pass through their bodies by diffusion. The digestive cavity has only one opening for both ingestion and waste removal. They have 2 hosts: a mammalian and an invertebrate intermediate host (freshwater snails) [18]. All Schistosoma (including SH) infects humans through direct skin contact with free-swimming larval forms of the parasite (cercariae) in freshwater. The cercariae penetrate the skin losing their bifurcated tails to become schistosomula entering the cutaneous venules and lymphatic vessels, traveling to the pulmonary vessels and then to the portal venous system, where they mature and unite the female in the gynecophoral canal of the male. The SH male/female pair find their way to the perivesical venous plexus and the veins draining bladder, prostate, and seminal vesicles. Eggs transfix the lumen of blood vessels into surrounding tissues passing through the bladder wall tissues and mucosa to shed in the urine. Nevertheless, many eggs are trapped in the bladder wall and release antigens and other metabolites causing the chronic features of urinary schistosomiasis through mechanical irritation, immunologic reactions, and their sequelae [19]. When urine of the infected person is voided in freshwater, the eggs hatch, releasing miracidia that, subsequently, infect the non-mammalian host (snail Bulinus species) where two asexual reproduction occurred, primary and then daughter sporocysts, and cercariae are released to re-infect a human and repeat the life cycle [20] (Fig. 1). Knowing the parasite life cycle, it is obvious that no infection of SH except with voiding of eggs from an infected person in freshwater that contains the specific snail to complete the life cycle. The produced cercaria can only re-infect another person whenever swim, bathe, or wade in polluted freshwater. Most of these factors are absent in developed countries.

### Schistosoma carcinogenesis: similarities and deviation from others

The International Agent for Research on Cancer (IARC) considered SH, *Opisthorchis viverrini*, and *Clonorchis sinensis* (the 3 parasitic agents) as group 1 definitive biological carcinogenic agents [21]. Several factors contribute to the oncogenesis of bilharzial infection. Many efforts were expended to determine the particular genes involved in this carcinogenic process. SH cell total antigen (worm extract) proved to cause urothelium cells to divide faster and died less frequently. This was related to increased levels of BcL-2 [22]. Dysplasia, low-grade intra-urothelial neoplasm, noninvasive malignant flat lesions, and bladder cancer are in 70% of mice upon single exposure of their urothelium to SH total antigen [23]. Furthermore, oncogenic mutation of the KRAS gene, possessing the carcinogenic ability, was detected in mice urothelium exposed to whole parasite extract [24]*.* In another series of studies, soluble egg antigens (SEA) extracted from Schistosoma egg positive urine proved the ability to enhance excessive proliferation, increase the oxidative stress (through reduced glutathione (GSH) depletion), and decrease apoptosis in cultured human prostate (PNT2) cells. Moreover, SEA was shown to diminish apoptosis in these cancer cells in a dose-dependent manner, by flow cytometric analysis with fluorescence staining [25]. Live eggs secret 2 proteins: IPSE and Omega-1-ribonuclease. These 2 proteins have overwhelming immunogenicity associated with glycan moiety and facilitating egg passage through the bladder wall but not resulting in fibrosis that immobilizes the egg, leading to egg-trapping [26]. Needless to mention that the ova deposited in the bladder wall initiate and aggravate intense inflammatory reactions, through the production of oxygen-derived free radicals. These free radicals are capable of producing genetic mutations and the production of numerous carcinogenic compounds (N-nitrosamines and polycyclic aromatic hydrocarbons) [25]. Normally, the urothelial (transitional) epithelium act as a natural barrier preventing urinary toxic materials from passive permeability through the wall. During chronic SH, toxic leakage can occur allowing for greater carcinogen entry [27]. The continuous deposition and retention of eggs in the bladder wall with subsequent traversal of the eggs through the urothelium results in tissue damage. The H-IPSE released from the eggs counteracts the urothelial damage by increasing cell proliferation leading to the development of bladder cancer [26].

Furthermore, p27 (cyclin-dependent kinase inhibitor 1B) inactivation and BcL2 upregulated expression were demonstrated to enhance cell proliferation and suppress apoptosis in Chinese hamster ovary (CHO) cells exposed to either SEA or whole parasites extract. The p27 is a tumor suppressor protein regulating the G1 phase of the cell cycle through inhibiting cyclin D and cyclin-dependent kinase 4 activation [28]. This inactivation results in unregulated and enhanced cell proliferation. On the other hand, BcL2 antagonizes the actions of proapoptotic proteins action. This may disturb the regularity mechanism of oncogenes and suppressor genes, stimulating the proliferative response to repair tissue damage caused by the inflammation [29].

The most common genetic change is the loss of heterozygosity (LOH) on chromosomes 9p and 9q, regardless of tumor grade or stage. There was no difference demarcating SA-BC from Schistosoma-non-associated BC (SNA-BC) in this loss of heterozygosity [20]. Nevertheless, it is known that BC is a cytogenetically heterogeneous disease, suggesting that pathogenesis may not be consistent for each case. The overexpression of the BcL2 gene in SA-BC patients was shown to be upregulated in squamous but not urothelial cancers. This explains the fact that BcL2 overexpression may lead to the predominance of SCC in SA-BC. Upregulation of BcL2 overrides programmed cell apoptosis, potentiates genomic instability, and interacts with various proto-oncogenes leading to carcinogenesis. BcL-2 overexpression was detected in 32%; TP53 mutations abnormalities were found in 73% of tumors while both TP53 and BcL-2 in 13%. Moreover, COX-2 overexpression was detected in SA-BC which has an active role in the complex multistage process of SA-BC carcinogenesis. Other mutations were detected including *H-RAS*, deletion of p16, and p15 increasing EGF receptor, c-ERB-2, and TNF-a. Prostaglandin overproduction upregulates COX-2, decreases killer T cell activity, and increases BcL2 and glutathione-*S*-transferase. These mutations may result in increased carcinogenicity through decreasing cell apoptosis and/or creating immunosuppression, adhesion molecule downregulation, degradation of the extracellular matrix, and enhancing angiogenesis [30]. The purified SH egg injected directly into mice bladder wall demonstrated urothelial hyperplasia, a potentially pre-cancerous lesion [31]. Microarray analysis revealed urothelial hyperplasia and decreased uroplakins transcript levels in mice bladder [32]. Moreover, the bladder dysplasia showed KRAS gene mutation demonstrating a link between the development of KRAS mutation and SA-BC [33].

Tumor suppressor gene TP53 dysfunction leads to tumor cell apoptosis and increased accumulation of p53 protein that correlates with tumor histological grade [34]. Nevertheless, p53 protein elevated level was documented in both pre-malignant and malignant lesions associated with schistosomiasis [32]. The increased p53 levels in these pre-malignant lesions likely resulted in TP53 mutations reported in bladder Schistosoma lesions. Furthermore, in another study, Chala et al. [35] administered both intact SH eggs and the cancer-inducing nitrosamine compounds (N-nitrosodimethylamine and N-butyl-N-(4-hydroxybutyl) nitrosamine) to the bladder mucosa and demonstrated that sub-carcinogenic levels of nitrosamines become carcinogenic when combined with SH intact eggs. This complementation finding provides further evidence to support the multi-hit hypothesis explaining urinary schistosomal carcinogenesis.

Epigenetic changes in the host genome may also have a role in SA-BC. The DNA derived from the urine SH-infected patients revealed hypermethylation of various genes. Furthermore, drug-induced inhibition of DNA methylation leads to decreased urothelium hyperplasia of the SEA-injected bladders [36].

Dysbiosis in the urine microbiome was also suggested as a risk factor for carcinogenesis [37]. A study of the urine microbiome from SH patients showed discrepancies in microbial communities between SH infected and uninfected subjects [38]. Adebayo et al. demonstrated various associations of the microbiome with different parameters, including the stage of bladder pathology and parasite infection burden. The combination of microbiome bacterial proteomics may facilitate the process of carcinogenesis [38]. Chronic schistosome infection, with changing microbiota, constantly secretes antigens changing the host microenvironment and may reveal pro-carcinogenic features. The combination of inflammation and mutagenic parasite antigens could explain the observation of increased chromosomal damage found in urogenital schistosomiasis patients [29].

Secondary bacterial infection, additionally, may lead to the production of the carcinogenic nitroso compounds (NOCs) via chemical reduction of nitrates by bacteria [39]. These NOCs resulted in DNA alkylation and mutagenicity. The chromosome 9p21-22 genomic alteration on the region encodes the major tumor suppressor genes. Additionally, this alteration results in p16 and p15 deletion more frequently in SA-BC than SNA-BC [40]. Moreover, genomic aberration on the Y-chromosome was found to be more frequent with SA-BC than SNA-BC [41]. Furthermore, SA-BC patients were found to have a significantly higher level of ANG (potent angiogenesis inducing protein) compared to non-SA-BC patients. The association between SA-BC and fibronectin level, E-cadherin in urine, and non-significant association with hyaluronidase, fibronectin release, and other ECM components is a common finding in SABC [42].

### Clinical manifestation of schistosomiasis

#### Acute stage

Acute schistosomiasis occurs only in infection-naïve, mainly travelers or immigrants to schistosome-endemic areas after exposure to schistosome antigens. Skin penetration by cercariae usually is not noticed by the patient, but can result in an itchy rash “swimmer’s itch.” Three-to-eight weeks after the initial infection, patients develop acute schistosomiasis syndrome (also called Katayama syndrome). This is a systemic hypersensitivity reaction to schistosome antigens and circulating immune complexes that coincide with the beginning of egg production. Sudden onset of fever, malaise, myalgia, headache, eosinophilia, fatigue, and abdominal pain are the typical presentation that lasts for 2–10 weeks. However, this presentation is lacking in residents of endemic areas, probably as a result of in-utero priming of T lymphocyte and B lymphocyte responses of babies born to mothers with chronic infection [43].

#### Chronic stage

*Schistosoma haematobium* (SH) infections present with bladder dysfunction and usually present as a chronic, gradually debilitating illness. Urinary SH affects the genitourinary organs including the bladder, lower ureters, seminal vesicles, and, less frequently, the vas deferens. The characteristic clinical presentation includes repeated attacks of terminal hematuria, dysuria, pyuria, dark urine, and/or increased frequency of micturition. However, it may remain asymptomatic for years [43]. Bladder function disruption or disturbance of the internal vesical or ureterovesical sphincter mechanisms may manifest. Diffuse fibrosis and scarring usually lead to a cicatricial contraction in the ureter, ureterovesical junction or urethra, obstructive manifestations (bladder neck obstruction), backpressure, and ascending infection. Furthermore, the association with bacterial coinfections including salmonella, tuberculosis, staphylococcus, *Neisseria gonorrhea*, *Chlamydia trachomatis*, *Mycoplasma genitalium*, and *Trichomonas vaginalis* may frequently occur [8].

### Pathological characteristics of urinary schistosomiasis and related cancer

Most of the pathological findings of schistosomiasis are secondary to inflammatory and immunological responses to egg deposition. The chronically infected bladder manifests as several abnormalities at the gross morphological and molecular levels. Pathologically, patchy or confluent fibrosis, as a result of healing granulomas, is associated with calcification that may interrupt the bladder and ureteric musculature. The Schistosoma histopathological lesions are classified into four stages: active granulomatous stage, chronic active stage, late residual stage, and chronic inactive stage depending upon the degree of inflammatory reactions, egg calcification, and extent of fibrosis [44]. Bladder mucosa shows inflammatory reactions with extrusion and penetration of the ova causing transient edema, hyperemia, and submucosal hemorrhage. Mucosal ulceration usually proceeds to a chronic ulcer as a result of secondary infection. The ulcers are mainly superficial with irregular sloping edges and a granular yellow floor devoid of the urothelium. The ulcer base shows sloughing tissue, viable or calcified eggs, inflammatory granulomatous tissue, and/or bands of fibrous tissues. These bladder lesions dry up, leaving pale mucosa with patches of a granular floor, known as “sandy batches [45]. Von Brunn’s nests, a pathognomonic for schistosomiasis, are grayish golden brown-colored proliferative lesions, either present within or separate from the mucosa forming, a well-defined solid structure within the lamina propria. This may be accompanied by squamous or less commonly adenomatous metaplastic changes. Cystitis cystica is a chronic reactive inflammatory disorder that commonly occurs in the trigone and presents as an irregular mamillated lesion with central eosinophilic liquefaction. Cystitis glandularis (glandular metaplasia) is similar to cystitis cystica except that the urothelial cells lining the cystic lesion have glandular metaplasia with goblet cells identical to those of the large bowel. Schistosoma tubercles are another specific lesion seen as multiple shiny yellowish or brownish elevated nodules and surrounded by a zone of hyperemia. These polypoid lesions may result from irritation of the mucosa by SH products. They appear as epithelial projection towards the bladder cavity. However, the basic and characteristic tissue reaction to Schistosoma eggs is the formation of Schistosoma (bilharzial) granulomata characterized by diffuse infiltration of eosinophilic response with abundant macrophages, multinucleated giant cells, and spindle cells surrounding the ova together with various amounts of fibrous tissues [44]. Although squamous cell carcinoma is the most common histology in SA-BC, other subtypes are represented with lesser frequency. The squamous category has a positive correlation with the intensity of Schistosoma infestation [7].

Histopathologically, bladder cancer is a heterogeneous group with at least 40 histological subgroups and is classified according to WHO 2016 [46] into:

A. Urothelial carcinoma (UC) that is the predominant histological type, mostly pure urothelial or less-frequent histological divergent differentiation (variants) including (a) squamous (SCC), (b) glandular differentiation (GD), (c) trophoblastic differentiation, (d) nested, (e) microcystic, (f) micro-papillary (MP), (g) sarcomatoid, (h) plasmacytoid/signet ring/diffuse, (i) giant cell, (j) poorly differentiated, lipid-rich, and (l) clear cell.
B. Squamous cell neoplasm: (a) pure squamous cell carcinoma and (b) verrucous carcinoma.
C. Adenocarcinoma: enteric, mucinous, mixed, and not otherwise specified (NOS).
D. Tumors of Mullerian type (clear cell carcinoma and endometroid carcinoma).
E. Mesenchymal tumors.

SA-BC has a higher percentage of squamous carcinoma and adenocarcinoma than NSA-BC [7, 46–48].

Generally, 75% of patients have non-muscle-invasive bladder cancer (NMIBC), whereas 25% belong to the muscle-invasive bladder cancer (MIBC) category. NMIBC is characterized by frequent recurrence (50–70%) but a low propensity to progress (10–15%) and a 5-year survival of 90%. On the other hand, MIBC is characterized by high rates of metastasis and a 5-year survival of < 50% despite radical surgery. SA-BC has a higher percentage of MIBC than SNA-BC [6]. However, distant metastasis in SA-BC was shown to occur in 23% of cases, a nearly similar rate to that of NSA-BC. This metastasis rate increased with high stage, high grade, and nodal involvement [49].

#### Diagnosis of urinary schistosomiasis

The diagnosis is confirmed by microscopic detection of eggs in urine. Serologic tests may be diagnostic especially in travelers with light infection. However, it does not differentiate current from past infection [7].

### Treatment of schistosomiasis

Modern chemotherapeutic drugs were introduced for the treatment of schistosomiasis in the late 1960s. Niridazole, hycanthone, and amoscanate showed some response, yet they were extremely toxic to prohibit its usage. Mitrofonate inhibits acetylcholine esterase and paralyzes the worm leading to its detachment from the venules. Subsequently, drug resistance was developed and stopped its use. Randomized clinical trials showed that isoquinoline drug “praziquantel,” a pyrazinoisoquinoline derivative, is a safe and effective oral drug that is active against all schistosome species. The drug is absorbed well from the gut and metabolized in the liver. Its metabolites are excreted in the urine. Laboratory studies showed that it causes tetanic contractions and tegmental vacuoles causing detachment of the worm from the walls of veins and subsequently die [49]. Praziquantel is most effective against the adult worm and mandates the presence of antibody response against the parasite. Furthermore, it may affect SH eggs lodged in tissue. For the traveler, praziquantel treatment should start at least 6–8 weeks post-exposure to potentially contaminated freshwater. Although a single course is usually curative in light infection, repeating treatment may be needed after 2–4 weeks to augment effectiveness [7]. Praziquantel is a cheap drug costing 0.2–0.3 US dollars per treatment course [49].

A multidisciplinary, integrated approach is the optimal goal for schistosomiasis elimination. It consisted of interventions, measures to control the source of SH infection, and integrated snail control. Public health interventions include improving sanitation and supply of clean water, snail control, and health education. Integrated snail control interventions using both biological and mechanical methods have shown promising results [50].

### Treatment of SABC

Superficial bladder cancer or NMIBC is treated with complete transurethral resection of bladder tumor (TURBT). Random biopsies from normal-looking mucosa are advisable for those with positive urinary cytology with no visible tumor. The American Urologic Association recommends enhanced cystoscopy at the time of TUR as the conventional white light cystoscopy inadequately detect all tumors. The enhanced cystoscopy techniques use either blue light cystoscopy or narrow-band imaging. The European Association of Urology recommends photodynamic diagnosis (PDD)-guided biopsies in case of negative cystoscopy or CIS-positive situations [51]. A second TURBT should be performed if the initial resection is incomplete. Adjuvant intravesical treatment depends upon risk group classification (low, intermediate, and high). It is recommended that one immediate intravesical chemotherapy instillation (mitomycin C, epirubicin, or doxorubicin) be performed to reduce the risk of recurrence in Ta, T1 tumors, with no superiority among these chemotherapeutic agents [52]. Immunotherapy (BCG) instillation is indicated in intermediate and high-risk tumors and is superior to chemotherapy for recurrence prevention. Although this treatment type is very popular in NSA-BC, it is less popular in SA-BC due to the prevalence of many bilharzial non-healthy mucosae, precancerous, or cancerous lesions in the bladder mucosa [47].

### Radical cystectomy

Radical cystectomy remains, for a long time, the treatment of choice in MIBC whether it is SA-BC or NSA-BC. The surgical procedure includes radical excision of the bladder, seminal vesicles, and prostate together with perivesical fat and peritoneal coverage, in addition to pelvic lymphadenectomy in male patients. In females, excision of the bladder, its perivesical fat and peritoneal coverage, urethra, uterus, ovary, and anterior wall of the vagina (anterior pelvic exenteration), is the usual procedure [53–55]. The overall and event-free survival of radical cystectomy showed similarity in SA-BC and NSA-BC when comparing stage by stage and when applying the same surgical technique [54–67] (Table 1).

**Table 1:** The 5-year overall survival rates by pathological stage in non-Schistosoma-associated bladder cancer (SNA-BC) and Schistosoma-associated bladder cancer (SA-BC)

Author	Year	Patients (*n*)	PT1	PT2	PT3	PT4	Nodal involvement
Non-Schistosoma-associated bladder cancer							
Stein et al. [55]	2001	1054	74	81/68^a^	47	44	35
Medersbacher et al. [56]	2003	507	76	62	40	49	26
Takahashi et al. [57]	2004	466	81	74	47	38	50
Nishiyama et al. [58]	2004	1113	82	84/69^a^	59	31.0	35
Shariat et al. [59]	2006	888	85.9	78.9	47.7	17	21.6
Dhar et al. [60]	2006	385	–	63	19	–	9
Gupta et al. [61]	2008	502	90	78	70/58^a^	46	–
Manoharan et al. [62]	2009	432	79	60	43	17	22
Lughezzani et al. [63]	2010	11,260	61	57	49	47	39
Schistosoma-associated bladder cancer							
Ghoneim et al. [64]	1997	1026	73	66	47/31^a^	19	23
El Mekresh et al. [65]	1998	185	83	–	41	–	21
Khaled et al. [66]	2005	180	55	–	12	–	6
Zaghloul et al. [67]	2006	192	100	100/47	40	44	31
Zaghloul et al. [68]	2007	216	100	51	40	30	31
Ghoneim et al. [54]	2008	2720	82	75/53^a^	40	30	27

^a^For stage a/b

Comparable results of adjuvant, neoadjuvant radiotherapy, or chemotherapy for SA-BC or NSA-BC were documented, when applying similar management protocols for the same stage or disease risk status [48]. A recent study on bladder cancer in the Schistosoma-endemic area showed that marginally improved results were obtained with a combination of adjuvant chemoradiotherapy in locally advanced stages [69]. This effect was statistically significant in Urothelial carcinoma but not squamous cell carcinoma [70].

### Bladder preservation tri-modality treatment

Bladder preserving tri-modality treatment is an alternative to radical cystectomy. It serves 3 goals: the eradication of the local disease, elimination of potential micro-metastasis, and maintaining the best possible quality of life (QoL) through urinary bladder preservation. Variable treatment protocols were carried out by different institutions; however, they comprised 3 essential procedures: maximal TURBT, to be followed by neoadjuvant chemotherapy or radio-chemotherapy; assessment cystoscopy for response evaluation; and determination of the following step. The third procedure is either consolidation radio-chemotherapy upon attaining complete response or radical cystectomy for those experienced less than complete response. Radical cystectomy remains a necessary procedure whenever there is an incomplete response or muscle-invasive recurrence [71, 72]. It is worth mentioning that there were no significant differences between the results in SA-BC and urothelial SNA-BC denoting that the association with schistosomiasis had no significant impact on the results of therapy for the bladder cancer patient.

## Conclusion

Urinary schistosomiasis is an endemic disease in many LMIC that may be encountered in immigrants from these countries. The SH carcinogenic mechanisms differ in some aspects than that in others. SA-BC is usually presented in more advanced stages due to the similarity of its symptoms to that of schistosomiasis which the patient used to have. The general outlines of management follow the same recommendations for bladder cancer (SNA-BC) and the clinical end-results are similar when compared stage by stage.

## Data Availability

Not applicable.

## Abbreviations

LMIC: Low-/middle-income countries
HIC: High-income countries
SA-BC: Schistosoma-associated bladder cancer
SNA-BC: Schistosoma-non-associated bladder cancer
WHO: World Health Organization
SH: Schistosomiasis
IARC: International Agent for Research on Cancer
GSH: Glutathione
SEA: Soluble egg antigen
CHO: Chinese hamster ovary
NOCs: Nitroso compounds
UC: Urothelial cancer
SCC: Squamous cell carcinoma
GD: Glandular differentiation
NOS: Not otherwise specified
MIBC: Muscle-invasive bladder cancer
NMIBC: Non-muscle-invasive bladder cancer
